# Supplementary material for: Reducing Sample Size While Improving Equity in Vaccine Clinical Trials: A Machine Learning-Based Recruitment Methodology with Application to Improving Trials of Hepatitis C Virus Vaccines in People Who Inject Drugs
Source: Healthcare (Basel). 2024 Mar 13;12(6):644. doi: 10.3390/healthcare12060644 (PMC10970332; doi:10.3390/healthcare12060644)
Supplement: Supplementary file 1 [file healthcare-12-00644-s001.zip › healthcare-2825382-supplementary.pdf]

Reducing Sample Size while Improving Equity in Vaccine Clinical Trials: a Machine Learning-Based Recruitment Methodology with Application to Improving Trials of Hepatitis C Virus Vaccines in People Who Inject Drugs

Richard Chiu, Eric Tatara, Mary Ellen Mackesy-Amiti, Kimberly Page, Jonathan Ozik, Basmattee Boodram, Harel Dahari, Alexander Gutfraind*
agutfraind@luc.edu

MAIN APPENDIX

Table of contents

SM. Supplemental Methods 2
- SM.A. HepCEP simulation model 2
- SM.B PREDICTEE strategy 4
- SM.C Modifications of the basic PREDICTEE scheme 8
- SM.D Extending PREDICTEE to the real-world setting 9

SR. Supplemental Results 12
- SR.A Baseline Characteristics 12
- SR.B PREDICTEE model prediction error and post hoc power analysis 13
- SR.C Effect of PREDICTEE targeting on subgroup incidence 15
- SR.D Binary feature targeting using PREDICTEE 17
- SR.E Effect of batch recruitment proportion on PREDICTEE 19

References 21

SM. Supplemental Methods

SM.A. HepCEP simulation model

The HepCEP model (formerly called APK) is an agent-based simulation model [1,2] which we used and extended as part of this study. HepCEP was first used in this study for creating a list of PWID in the Chicago area and was used secondly to simulate HCV infection events during a clinical trial. Below, we briefly describe the data and design of the model.

SM.A.i. PWID population data sources

Details on the generation of synthetic PWID via HepCEP were previously described in [1]. In brief, each HepCEP simulation contains a synthetic profile of each of the estimated 32,000 PWID [3] residing in metropolitan Chicago. The initial PWID value can also be set higher, increasing the total number of synthetic PWID generated. The generation is based on two empirical datasets licensed to the team: (i) 2009 metropolitan Chicago PWID data from the CDC-sponsored National HIV Behavioral Surveillance survey of 545 PWID [4] [NHBS 2009] and (ii) 2006-2013 data from large, multi-site syringe service program (SSP) of >6,000 participants [5] [CNEP]. To ensure accuracy, the synthetic population also contains under-studied suburban PWID, who make up an estimated 54% of the metropolitan Chicago PWID population (**Table 1**) [6]. Synthetic PWID are generated via random sampling of the >6,000 real PWID underlying HepCEP and stochastically adjusting age. Based on our analyses, an increased value of initial PWID does not result in a statistically significant difference in the characteristics of the starting population.

SM.A.ii. Geographic environment and network formation

The metropolitan Chicago model geography is defined by zones based on the 2010 US Census ZIP code area data. Geographic locations of importance to PWID (residence and known drug market locations) from the two empirical datasets used to generate the synthetic population were embedded into the metropolitan Chicago geographic environment. Syringe- sharing was modeled as the primary mode of HCV transmission, and PWID are connected via syringe-sharing networks. Network formation was determined by the probability of two persons encountering each other in their neighborhood of residence or within known drug market areas in Chicago, Illinois. The markets attract both urban and non-urban PWID for drug purchasing and utilization of SSPs that are also located in the same areas [6]. The methods used to calculate network encounter rates, establishment processes, and removal of networks are detailed in [1]. Each individual has a predetermined number of in-network PWID partners who give syringes to the individual

and out-network predetermined PWID partners who receive syringes from the individual, which drives the direction of HCV transmission. The network is dynamic, and during the course of simulation, some ties may be lost while new connections form, resulting in an approximately constant network size. PWID agents can leave the model population either due to age-dependent death or permanent drug use cessation and are replaced with new agents sampled from the input data set to maintain a nearly constant population size of 32,000, or another user-set value, for the entire course of the simulation. The annual turnover rate of the population is about 2%.

SM.A.iii. Population validation

Two empirical datasets were obtained on metropolitan Chicago PWID to validate HepCEP. The 2012 NHBS Chicago PWID subset, the most representative dataset available at the time, was used to construct a synthetic population to validate HCV prevalence for 2012. The previous validation results show high concordance, i.e., the predicted and actual values match within 2% overall for HCV prevalence [1]. Similarly, data from a 2012-13 network and geographic study of 164 PWID ages 18-30 and their drug-using network members [6] were used to calibrate and validate the network formation process. The simulated and actual networks match closely with an average error of 1.3% [1,7].

SM.B PREDICTEE strategy

SM.B.i. Background and Similar Research

The underlying inspiration of this recruitment strategy is the concept of prognostic enrichment, which seeks to recruit a study cohort with a higher rate of a clinical event than the broader population. The use of prognostic enrichment in clinical trials is not a new concept, with multiple previous studies illustrating the clinical and statistical benefits it can provide [8–10]. In the context of an HCV vaccine trial, prognostic enrichment will serve to enroll participants who are more likely to become infected with HCV, resulting in a more detectable primary outcome. This will also ease resource costs associated with recruitment since a higher incidence implies a smaller required sample size, and it is more ethically responsible as fewer people are exposed to the experimental drug.

PREDICTEE could be seen as contributing to the literature on online optimization algorithms, particularly the so-called secretary problem [11]. However, unlike the secretary problem that seeks to find a single optimal person, our goal with vaccine RCTs is to create a high incidence and representative cohort of "secretaries." Additionally, there has recently been a lot of concern in the literature about under-representation of racial and ethnic minorities in clinical trials [12,13], as well as the need to ensure the generalizability of trials [14].

SM.B.ii. Use of Synthetic Population Dataset

Our synthetic population of 123,071 PWID is based on real survey data of >6,000 PWID in Chicago. Each of these real PWID are assigned a database ID (DBID), and synthetic PWID are generated via stochastic modification of these PWID. Each of these synthetic PWID are assigned a DBID corresponding to the real PWID they are generated from. To prevent any information leakage during training and testing of the model in our simulations, we split the 123,071 profiles into training and test sets based on the DBID of the CNEP participant used in generating the synthetic profile.

SM.B.iii. Parameters of PREDICTEE

The parameters of the PREDICTEE strategy are listed in **Table S1**.

Table S1. PREDICTEE parameters, values used in our simulations, and recommended ranges.

Parameter	Definition	Description	Initial value	Range
B	Batch size	Number of candidates received from the outreach process each step	50	[20-100]
R	Recruited per step	Number of recruits for each time step (out of the batch or backlog)	5	[1-25]
N	Sample size	Number of subjects needed, assuming initial incidence estimates	800	Optionally re-estimated during run
W	Total work	Number of candidates considered (equivalently, time allowed for recruitment)	8000	*S* * (*B*/*R*)
w_0	Initial weight of incidence	Initial weight assigned to incidence	100, decreased by Δw each batch	Fixed
w_{min}	Incidence weight minimum	Lower bound of incidence weight	25	[0-50]
T	Trial follow-up period	The duration, in years, of the trial follow-up period after vaccination	1.5	[1-3]
v	Expected vaccine efficacy	The expected vaccine efficacy of the experimental agent	0.6	[0-1]
p	Demographic composition of the target population	Demographic breakdown of each category j of the target population.	Matrix/vector of proportions describing the target population	
C	Incidence model	Predictive model (often Cox model) indicating the probability of HCV infection during a trial		A Cox or RSF model
E	Re-estimation point	Specifies the point in the trial (after E candidates recruited) when sample size re-estimation occurs	Cox: 400<br>RSF: 267	Depends on predictive model efficacy
L	Backlog attrition probability	Probability after each batch that any given agent in the backlog is removed via attrition	0.2	[0-1]

SM.B.iv. Candidate Scoring

PREDICTEE candidate scoring is outlined in the Methods section of the main manuscript, specifically in Eqn. 1. To clarify this process, we offer a scoring example in **Fig. S1**, considering a microbatch of three candidates and assuming an existing partial cohort of ten enrollees. Ideally, in an actual RCT, batch size *B* should have a larger value (>20). In this example, we seek to ensure two demographics are representative of the target population: race and sex.

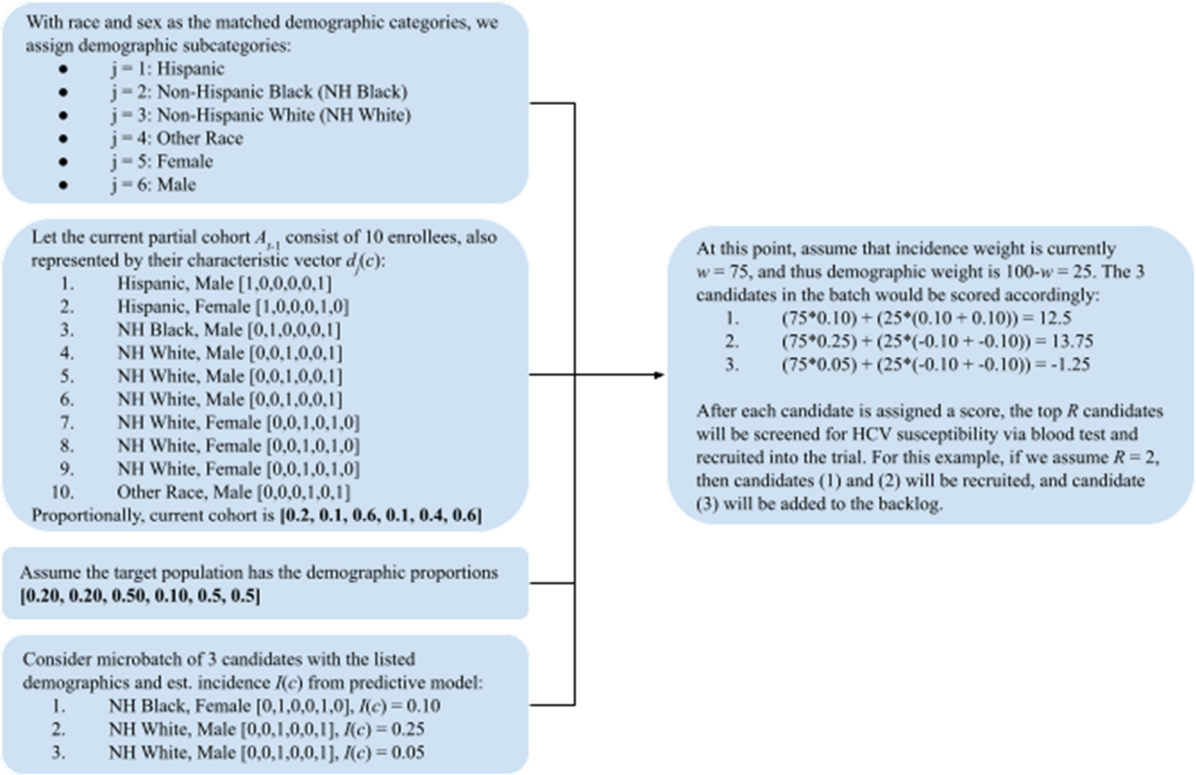

Figure S1. An example of the scoring process used in PREDICTEE.

As noted in the main text, the performance of the scheme would be maximized if the study site developed its own predictive model of incidence, but we realize that it is not feasible in every situation. In those cases, several strategies could be implemented: to use our Chicago-area model (if the site is sufficiently similar to Chicago) or other existing models, or to build a simple heuristic model where candidates are given points for any factor that is likely to increase the incidence. Because incidence cannot be robustly estimated for the cohort, early stopping of the trial should be used more sparingly.

Additionally, all sites would be well-advised to monitor the recruited candidates continuously and detect any unexpected biases as might occur. Both with conventional recruitment and with PREDICTEE, the field team might observe that the recruited candidates misrepresent the population along criteria not anticipated during the study design or the design of the scoring system. With PREDICTEE, it is possible to adjust the scoring system during the trial to correct the cohort and remove such a bias.

SM.B.v. Sample size estimation and re-estimation

Group sample size calculations were performed using the following formulae [15]:

$$N = 4\left(\frac{Z_{1-\beta} + Z_{1-\alpha}}{h}\right)^2 \quad \text{and} \quad h = 2\ arcsin\ (\sqrt{P_1}) - 2\ arcsin\ (\sqrt{P_2})$$

where $1\text{-}\beta$ is power, α is the significance level, and P_1 and P_2 are the proportion of the respective trial arm that is expected to develop an HCV infection. Throughout this study, group sample sizes are calculated assuming 80% power, a 95% confidence level, and a 60% vaccine efficacy [16].

In this study, blinded sample size re-estimation is used alongside prognostic enrichment to greatly reduce the required sample size for a vaccine trial while also minimally impacting the type I error rate [17]. The benefits of sample size re-estimation are well documented in the literature [17,18]. In scenarios where cohort incidence of the disease based on interim analysis is different than what was expected in the initial sample size calculations, the sample size can be adjusted upwards or downwards based on statistical needs. Re-estimation also confers ethical and practical benefits in scenarios where the sample size is adjusted downwards, since fewer people would be exposed to the drug and fewer participants would need to be recruited. In a real trial, the process of sample size re-estimation would be under the purview of the clinical trial data safety and monitoring board.

SM.C Modifications of the basic PREDICTEE scheme

SM.C.i. High demographic gap

The weight adjustment process is outlined in the Methods section of the main manuscript, specifically in Equation 2. As mentioned, for situations where the target population is greatly dissimilar to the population available for recruitment, PREDICTEE includes an optional parameter that adds an additional adjustment to the weights which would prioritize demographics, represented by χ in Eqn. 2a. The succeeding term represents the greatest absolute difference between a demographic proportion of the target population and the partial cohort, and it is designed so that it will converge to 0 as the representation of the trial cohort improves, gradually diminishing the importance of this second half of the equation. In most practical scenarios, the value of χ will be set to 0.

$$w_t = w_{t-1} - \frac{R}{N}(w_{t-1} - w_{min}) - \chi(max_{j\in J}[p_j - \frac{1}{|A_{t-1}|}\sum_{a\in A_{t-1}} d_j(a)]) \quad (2a)$$

We set $\chi = 1$ when running the simulations of PREDICTEE recruitment, with the target being set to an arbitrary maximally balanced population with an equal distribution of race and sex demographics. It should also be noted that w_t may never fall below the incidence weight floor w_{min}, and we assign $w_t = max(w_t, w_{min})$.

SM.C.ii. Backlog of Candidates

In PREDICTEE, we also explored the value of maintaining a backlog—a list of candidates who were not immediately recruited—and found that it has a positive but small effect. We hypothesize that when the batch size is fairly large (>20), it is common to see some good candidates in each batch, and the backlogged candidates are rarely recruited. The backlog is probably most effective in settings when the number of candidates available in each batch is small or varies from week to week. When that occurs, the backlog provides a supply of good-quality candidates when none have arrived recently. We also found that there is little harm caused by dismissing from the backlog of candidates who were not recruited after five or so batches, as they are unlikely to ever be recruited. Each study site should carefully consider its conditions when calibrating these policies.

SM.D Extending PREDICTEE to the real world setting

In our main manuscript, we describe the PREDICTEE workflow and how we implemented it in a simulated trial setting. In this section, we describe how we foresee PREDICTEE being applied in a real-world setting. A general overview of the real-world workflow for PREDICTEE is detailed in **Figure S2**.

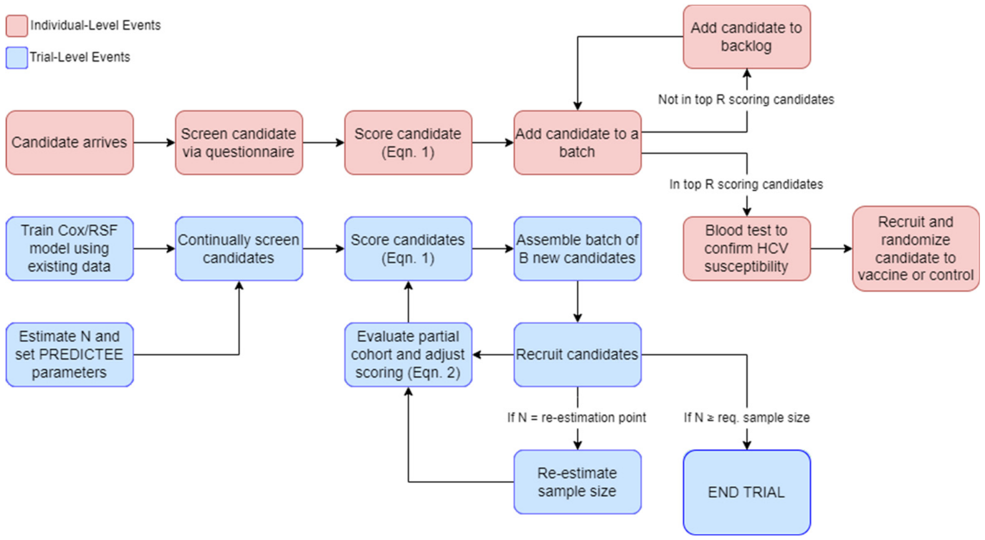

Figure S2. A schematic of PREDICTEE recruitment as it would occur in the real-world setting, divided into individual-level events (what an individual PWID would experience) and trial-level events (what trial coordinators would organize).

SM.D.i. Data Source

The basis of our simulated trials was the synthetic dataset generated by the HepCEP model. A portion of these data were used to train the survival analysis models, and the remainder served as the recruitment pool from which PWID were recruited. In a real trial, coordinators would simply need sufficient data to train an adequately powered survival analysis model. We foresee this coming from one of two sources: (1) a representative synthetic population using a model such as HepCEP, similar to what was carried out in our simulations, or (2) collecting longitudinal data of local PWID, which would only be feasible in sites with a large, established PWID population.

SM.D.ii. Setting PREDICTEE Parameters

In the field, investigators could determine the value of R (number recruited per batch) with the following calculation. First, an initial sample size S is determined based on statistical power considerations before PREDICTEE is initiated based on existing data on the study site. The investigators then estimate the total

number of candidates they can screen during the trial, W. If the investigators plan to process B candidates per time step ("batch"), the trial will have $z = W/B$ batches, and each will recruit an average of R candidates. To achieve its sample size, the value of R must be adjusted so that $R = S/z$.

SM.D.iii. Screening

In our PREDICTEE simulations, screening was simulated by a randomly sampling B candidates from a recruitment pool of synthetic PWID. In the real-world setting, PREDICTEE would likely work with a variety of outreach processes and inclusion and exclusion criteria. For example, outreach could involve contacting PWID with known contact information, using word-of-mouth, incentivizing network promotion, incorporating online promotion, or other strategies. The nature of the outreach process is expected to affect the demographics and incidence of the stream of candidates, and PREDICTEE will then shape recruited candidates to meet the trial's target composition and maximize the incidence. Interested candidates are expected to complete a screening questionnaire, which would determine eligibility and provide inputs to the predictive model by asking candidates about their demographic characteristics, network characteristics, and injection behavior. Of course, any data collection and participation should be with full disclosure and the informed consent of the participant.

SM.D.iv. Alternatives to Batchwise Enrollment

As described in our Methods, PREDICTEE recruits candidates in batches of B and enrolls the top-scoring candidates. Batch recruitment has been used in previous clinical trials [19,20], particularly in cases where observation time may be needed between subsequent enrollments [21]. The advantage of this process is that it frees the investigators from the perilous task of optimally setting a fitness threshold for candidates. It allows the trial to continuously locate and enroll candidates as the batch is filled rather than suffer gaps in recruitment when the arriving candidates have insufficient scores.

We considered an alternative scheme of *continuous recruitment* that could work in some settings and may be easier to implement in the real world: setting a numerical threshold and advancing any candidate whose score exceeds the threshold (subject to serological tests). Continuous recruitment allows faster recruitment in some settings but suffers from several pitfalls. First, setting a static score threshold is risky because mistakes based on limited initial information may result in under-recruitment or over-recruitment of candidates if the threshold is not correctly set. Seemingly, it is possible to adjust the threshold in real time, but that might require a complex optimization process to correctly do so under the uncertainty about the quality of the arriving candidates. Batchwise enrollment does not face this issue because a predetermined number of candidates R will be recruited at every batch. Second, the batch method is more

compatible with the cadence of practical RCTs, which involves multiple processing and testing steps. Therefore, batch recruitment should be preferred when there is uncertainty regarding and variability in the quality of the candidates and when attrition due to the associated delay in recruitment is small.

SM.D.v. Confirming HCV Susceptibility

Following screening, scoring, and selection of top-scoring candidates in our PREDICTEE simulations, only HCV-naïve PWID (RNA/antibody-negative) are recruited to the trial cohort. In the real setting, this would require the use of a blood test that is administered immediately after candidate scoring. This ordering (scoring first via questionnaire responses followed by a blood test) comes with the advantage of cost savings by requiring blood tests on just a small proportion (B/R) of the candidates who are invited to enroll. Additionally, only one test is required; there is no need to screen and retest the candidate again to confirm that the candidate remains uninfected just prior to enrollment. This order also simplifies the process by ensuring all candidates first answer the questionnaire and enter the predictive model. Alternatively, blood testing can occur concurrently with the questionnaire, and the predictive model is run afterwards. While this ordering comes with the disadvantages of requiring blood tests on every candidate and potentially needing to redo blood tests prior to enrollment, this may be necessary in trials where the recruitment needs to stratify (or represent) different serotypes and genotypes, or when the HCV positivity rate is relatively high.

SR. Supplemental Results

SR.A Baseline Characteristics

To gain insight into characteristics that PREDICTEE targets, we calculated the baseline characteristics of the PWID who were recruited in the 10,000 simulated trials using Chicago's susceptible PWID population as the target population and compared them to those of the random recruitment and in-network recruitment populations (Table S2). Notably, PREDICTEE tended to target PWID who were not enrolled in a harm reduction (HR) program, had a greater probability of receptive sharing, and who received drugs or injection equipment from other PWID. In other characteristics that were considered in the training of the Cox and RSF model, such as number of daily drug injections, there was not a significant increase in the cohorts recruited by PREDICTEE compared to conventional recruitment.

Table S2. Baseline characteristics of random recruitment and in-network recruitment populations compared to PREDICTEE-recruited cohorts. IQR: interquartile range

**Attribute**	**Random Recruitment**	**In-Network Recruitment**	**Cox PREDICTEE**	**RSF PREDICTEE**
Demographic attributes				
Location (by ZIP Code)	City: 36.5%<br>Suburbs: 63.5%	City: 27.5%<br>Suburbs: 72.5%	City: 25.2%<br>Suburbs: 74.8%	City: 32.8%<br>Suburbs: 67.1%
Race/ethnicity	Hispanic: 18.1%<br>NH Black: 15.5%<br>NH White: 63.2%<br>NH Other: 3.2%	Hispanic: 17.5%<br>NH Black: 10.0%<br>NH White: 69.3%<br>NH Other: 3.2%	Hispanic: 18.0%<br>NH Black: 15.1%<br>NH White: 63.4%<br>NH Other: 3.5%	Hispanic: 18.1%<br>NH Black: 15.4%<br>NH White: 63.1%<br>NH Other: 3.4%
Sex	Female: 31.5%<br>Male : 68.5%	Female: 37.8%<br>Male : 62.2%	Female: 31.9%<br>Male: 68.1%	Female: 31.8%<br>Male: 68.2%
Age, mean (IQR)	31.4 (24.9-37.0)	29.8 (24.0-34.0)	31.0 (24.2-36.5)	30.9 (24.0 - 36.7)
Enrollment in any HR program	HR: 45.5%<br>Non-HR: 54.5%	HR: 33.1%<br>Non-HR: 66.9%	HR: 10.5%<br>Non-HR: 89.5%	HR: 28.3%<br>Non-HR: 71.7%
Behavioral and network attributes				
Daily injections, mean (IQR)	2.4 (0.8-3.2)	2.6 (0.9-3.6)	2.9 (0.8-4.0)	2.7 (1.0-3.8)
Probability of receptive sharing, mean (IQR)	21.3% (0.0%-40.6%)	30.3% (5.0%-50.0%)	45.4% (32.8%-59.7%)	32.7% (9.2%-51.5%)
In degree (receptive network size)	0 (no network) - 66.0%<br>1 - 25.8%<br>≥2 – 8.3%	0 (no network) - 0%<br>1 - 75.7%<br>≥2 – 24.3%	0 (no network) - 51.1%<br>1 - 34.2%<br>≥2 – 14.9%	0 (no network) - 30.1%<br>1 - 51.1%<br>≥2 – 18.8%
Out degree (giving network size)	0 (no network) - 69.4%<br>1 - 22.7%<br>≥2 - 7.9%	0 (no network) - 45.0%<br>1 - 40.5%<br>≥2 - 14.5%	0 (no network) - 56.1%<br>1 - 32.3%<br>≥2 - 11.6%	0 (no network) - 48.6%<br>1 - 37.1%<br>≥2 - 14.3%

SR.B PREDICTEE model prediction error and post hoc power analysis

Given the importance of predicted incidence in sample size re-estimation of PREDICTEE, we also checked for any differences between the predicted incidence given by the trained model and the observed incidence of the recruited cohort during 1.5 years of the trial. To ensure the results are consistent, we compared the predicted and observed incidence in 10,000 trials. For both the Chicago susceptible and the arbitrary target populations (**Table S3** and **Table S4**, respectively), the differences in incidence were not statistically significant, with overlapping 95% ranges. There was also a tendency towards overprediction by 5-10% that appears to be slightly stronger in **Table S4**. Using the cohort incidence, we also calculated the post hoc power to assess for potential loss in statistical power as a result of PREDICTEE. The results show a minimal decrease from the target power of 80%, with RSF PREDICTEE retaining the most power after recruitment. However, the power loss increases as the amount of required demographic adjustment increases, as shown by comparing **Table S4** to **Table S3**.

Table S3. Predicted incidence compared to observed cohort incidence and post hoc power for PREDICTEE recruitment simulations when the demographic target is set to Chicago susceptible PWID. Values represent the mean of 10,000 simulations with 95% ranges calculated using quantiles.

	PREDICTEE (Cox)	PREDICTEE (RSF)
Cohort Incidence	.097 (.090–.104)	.149 (.141-.155)
Predicted Incidence	.101 (.083–.121)	.160 (.142–.174)
Post Hoc Power	76.3% (73.5%–79.4%)	79.1% (77.1%–80.9%)

Table S4. Predicted incidence compared to observed cohort incidence and post hoc power for PREDICTEE recruitment simulations when the demographic target is set to an arbitrary target that varies greatly from the recruitment pool. Values represent the mean of 10,000 simulations with 95% ranges calculated using quantiles.

	PREDICTEE (Cox)	PREDICTEE (RSF)
Cohort Incidence	.085 (.079–.095)	.137 (.130–.144)
Predicted Incidence	.095 (.075–.117)	.148 (.136–.165)
Post Hoc Power	71.4% (68.4%–75.7%)	75.8% (73.7%–77.9%)

Detailed investigation found that several benign factors caused this difference in incidence: (1) overcontribution of HCV-infected PWID to the predictive models, (2) differences in the time horizon in training and in the trial horizons, and (3) the choice of a low recruitment proportion (R/B), as follows. The training of survival models is highly influenced by cases of PWID that experience infections during the monitoring window (in our case, 10 years of simulation). Indeed, we confirmed that decreasing case weights in RSF for PWID that experienced HCV infection resulted in a reduction in prediction error. Case weights are essentially values attributing greater importance to certain records in the training data, thus implying that some overprediction may be due to HCV-infected PWID having too strong of an effect on the predictive model. Since the specific weights assigned to individual cases largely depend on the trial design and we sought to construct a trial-agnostic model, these weights were not included in the training; however, users interested in PREDICTEE may tune these weights according to their own needs and data resources to produce a more accurate predictive model. Additionally, the synthetic dataset was derived from a 10-year simulation with HepCEP, resulting in event times and censoring times for PWID that ranged up to 10 years. However, our simulated trials only predicted HCV status after a 1.5-year trial, leading to a horizon difference that partly contributes to overprediction, and we found that it could be reduced by adjusting the training. Lastly, as shown in error bars in **Figure S5**, a lower batch recruitment proportion resulted in increased prediction error in the resulting cohort. Thus, our choice of a 0.1 recruitment proportion for our simulations led to increased prediction error. This may be a consequence of higher-risk PWID being recruited into the trial, and since there are fewer data on these PWID, prediction of incidence may be less accurate.

SR.C Effect of PREDICTEE targeting on subgroup incidence

We investigated whether preferring certain underrepresented population categories might reduce their incidence in the recruited cohort. We compared weighted PREDICTEE, in which both infection risk and demographics are included in the recruitment decision, against unweighted PREDICTEE, in which only incidence is considered in recruitment. These results are reported in **Fig. S2**. In all of the race, sex and age categories (except 49+ with Cox PREDICTEE weighted), PREDICTEE either increased the mean incidence as compared to in-network recruitment or was comparable to in-network within a 95% range. We found that the effect of weighting on incidence depends on the abundance of the population category in the available population. For demographics such as non-Hispanic black, male, and age >49 where the proportion in the recruitment pool is lower than the proportion in the target population, PREDICTEE with weighting leads to an incidence lower than if there was no weighting, but a still greater incidence on average than with conventional recruitment. Trials that target a demographic which is disproportionally rare in their outreach (feeder) process should monitor the incidence in the targeted population and refine their outreach strategy.

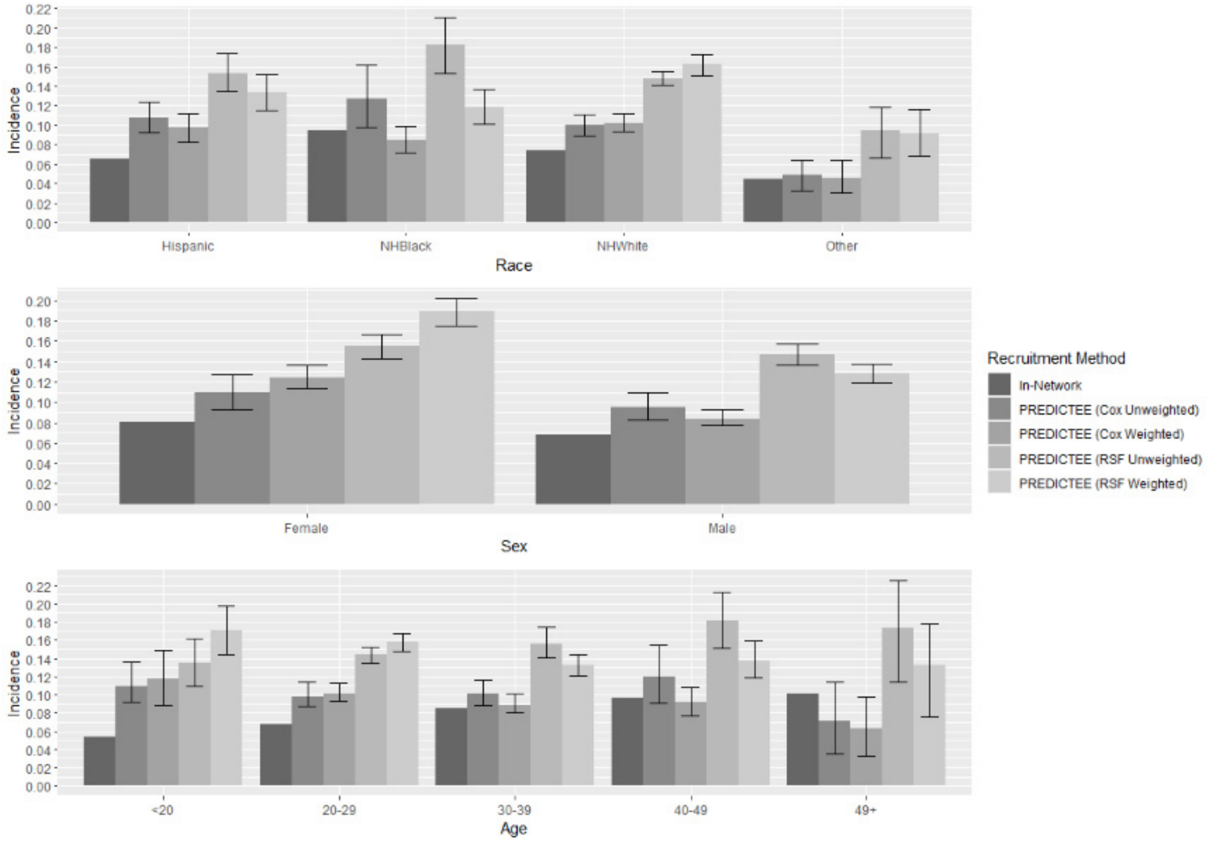

Figure S3. Comparison of observed cohort incidence within demographic subcategories for in-network recruitment as well as unweighted (only incidence considered) and weighted (both incidence and

demographics considered) PREDICTEE. Each bar represents the mean of 100 simulations. In-network recruitment values were calculated from the recruitment pool by determining the cumulative incidence of all susceptible PWID with a receptive network.

SR.D Binary feature targeting using PREDICTEE

While most of our trials only adjusted for up to three variables (race, sex, and age), we also added simulations that assessed PREDICTEE performance with up to 20 binary features, which could represent HCV genotypes, comorbidities, and other important prognostic factors in a real clinical trial. To do this, each candidate in the recruitment pool was randomly assigned a binary value for each of 20 features. Values were weighted so that the approximate composition for each feature was skewed 25:75 ("0" : "1"). Then, 100 simulations were run for a progressively larger number of matched binary categories from 0 to 20. This was performed for both Cox and RSF PREDICTEE, with the target composition of each feature being 50:50. For all simulations, we kept the parameter values as R = 5 and B = 50 with a desired power of 80%.

The results of these simulations are shown in **Figure S3**. Our results illustrate that on average, across 100 simulations, cohort incidence decreases, prediction error remains relatively unchanged, as desired, but PPR_{min} will decrease as more features are matched by PREDICTEE. Cox PREDICTEE was superior on average to in-network recruitment in terms of incidence when fewer than eight binary features were matched. The RSF version was superior on average to in-network recruitment even when twenty features were matched.

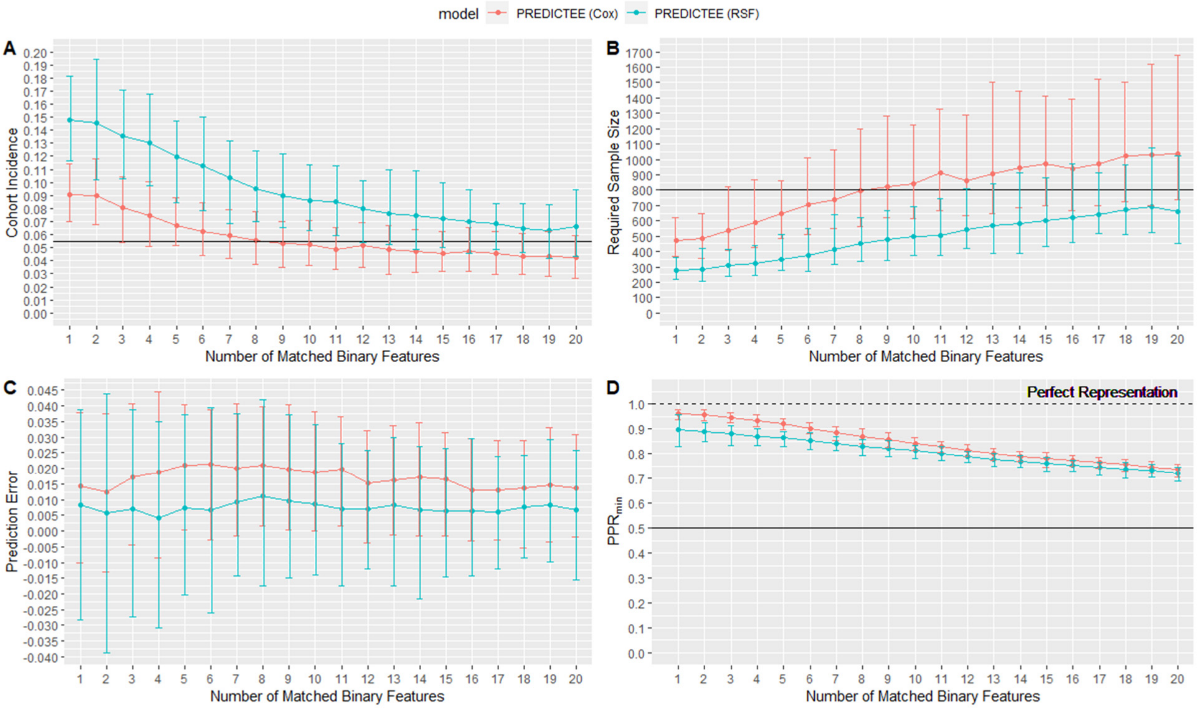

Figure S4. Performance of PREDICTEE in terms of (A) cohort incidence, (B) required sample size, (C) prediction error, and (D) PPR_{min} when recruitment must match binary features such as genotypes. Each data point represents the average of 100 simulations, with intervals representing 95% of simulations. In (C), positive and negative values represent over- and underprediction, respectively. The solid black line in (A) and (B) represents the corresponding average value for simulated cohorts using in-network recruitment. No black line is present in (C) because no incidence prediction is involved in the in-network recruitment method. The black line in (D) is placed at 0.5 because we assume that all binary features are randomly distributed and independent of drug-sharing behavior. Thus, in-network recruitment would, on average, result in a PPR_{min} of 0.25/0.50 = 0.5.

SR.E Effect of batch recruitment proportion on PREDICTEE

We also explored the effect of recruitment proportion by varying the batch recruitment proportion R/B systematically. We expected that increasing R/B would reduce incidence and increase sample size but were uncertain on its effect on representativeness. **Figure S4** illustrates the results of these simulations. Cohort incidence decreases as recruitment proportion increases, translating to an increase in sample size. RSF outperformed Cox PREDICTEE in terms of incidence, although the benefits were eliminated as recruitment proportion increased above 0.25. Compared to in-network recruitment, both Cox and RSF PREDICTEE were superior in terms of incidence/sample size at or below a recruitment proportion of 0.25 (**Figure S4A and S4B**). With regard to accuracy of the models used in PREDICTEE, error between predicted and cohort incidence was greater at lower recruitment proportions, especially for Cox PREDICTEE (**Figure S4C**). PREDICTEE, on average, outperformed in-network recruitment in terms of PPRmin at all recruitment proportion levels. As recruitment proportion increased, PPRmin increased, with large increases seen at the 0.05 and 0.10 levels (**Figure S4D**).

The prediction error for RSF PREDICTEE remained relatively constant and low at all recruitment proportions; however, the prediction error for Cox PREDICTEE spiked considerably at lower recruitment proportions, possibly due to candidates who experience HCV infection having too strong of an effect on model training (see Appendix SR.B). RSF was less prone to this effect, given that tree-based models are more resistant to outliers compared to regression-based model [22]. Our results suggest that PREDICTEE implementing a RSF model with lower recruitment proportions is most desirable, given its superiority in reducing sample size and screening requirements while also maintaining similar values of PPR_{min} and prediction error as Cox PREDICTEE.

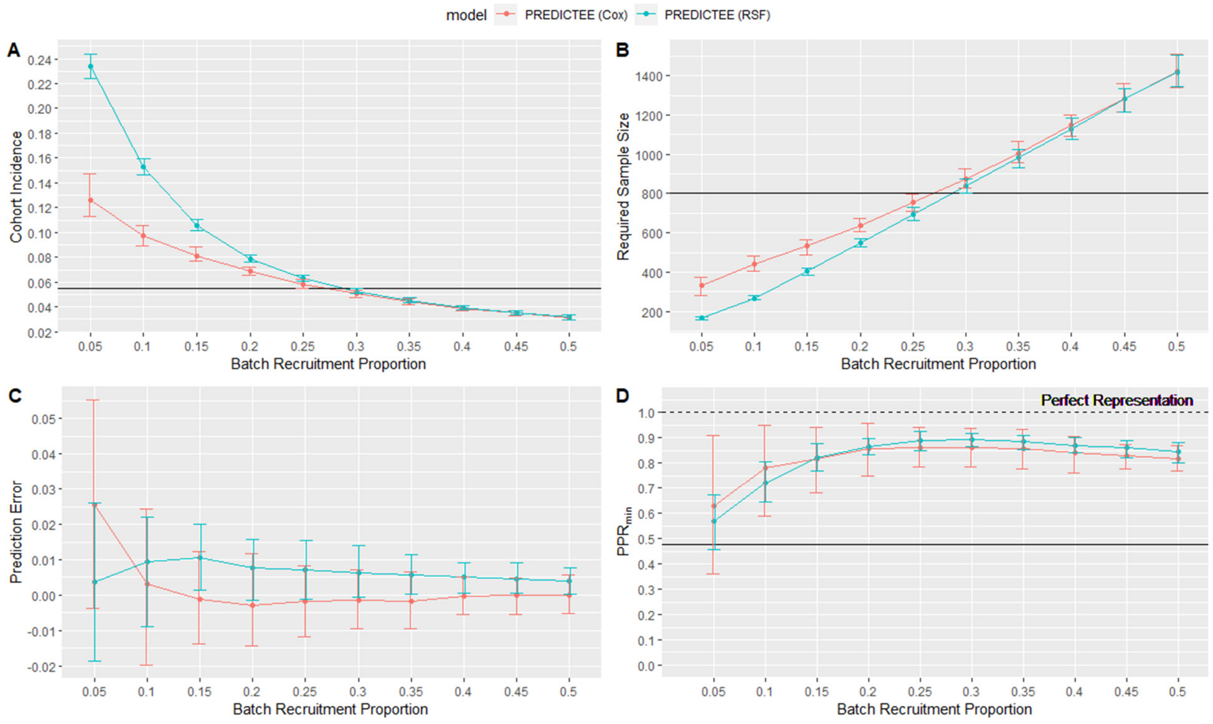

Figure S5. Performance of Cox and RSF models on different batch recruitment proportions in terms of (A) cohort incidence, (B) required sample size, (C) average prediction error, and (D) PPR_{min}. Each data point represents the average of 10,000 simulations, with intervals representing 95% of simulations. The solid black line in (A), (B), and (D) represents the corresponding average value for simulated cohorts using in-network recruitment, and it is not present in (C) because no incidence prediction is involved in the in-network recruitment method.

References

1. Gutfraind, A.; Boodram, B.; Prachand, N.; Hailegiorgis, A.; Dahari, H.; Major, M.E. Agent-Based Model Forecasts Aging of the Population of People Who Inject Drugs in Metropolitan Chicago and Changing Prevalence of Hepatitis C Infections. *PLOS ONE* **2015**, *10*, e0137993, doi:10.1371/journal.pone.0137993.
2. Tatara, E.; Collier, N.T.; Ozik, J.; Gutfraind, A.; Cotler, S.J.; Dahari, H.; Major, M.; Boodram, B. Multi-Objective Model Exploration of Hepatitis C Elimination in an Agent-Based Model of People Who Inject Drugs. *Proc Winter Simul Conf* **2019**, *2019*, 1008–1019, doi:10.1109/wsc40007.2019.9004747.
3. Tempalski, B.; Pouget, E.R.; Cleland, C.M.; Brady, J.E.; Cooper, H.L.F.; Hall, H.I.; Lansky, A.; West, B.S.; Friedman, S.R. Trends in the Population Prevalence of People Who Inject Drugs in US Metropolitan Areas 1992–2007. *PLOS ONE* **2013**, *8*, e64789, doi:10.1371/journal.pone.0064789.
4. Lansky, A.; Abdul-Quader, A.S.; Cribbin, M.; Hall, T.; Finlayson, T.J.; Garfein, R.S.; Lin, L.S.; Sullivan, P.S. Developing an HIV Behavioral Surveillance System for Injecting Drug Users: The National HIV Behavioral Surveillance System. *Public Health Rep* **2007**, *122*, 48–55, doi:10.1177/00333549071220S108.
5. Huo, D.; Ouellet, L.J. Needle Exchange and Injection-Related Risk Behaviors in Chicago: A Longitudinal Study. *JAIDS Journal of Acquired Immune Deficiency Syndromes* **2007**, *45*, 108–114, doi:10.1097/QAI.0b013e318050d260.
6. Boodram, B.; Hotton, A.L.; Shekhtman, L.; Gutfraind, A.; Dahari, H. High-Risk Geographic Mobility Patterns among Young Urban and Suburban Persons Who Inject Drugs and Their Injection Network Members. *J Urban Health* **2018**, *95*, 71–82, doi:10.1007/s11524-017-0185-7.
7. Boodram, B.; Mackesy-Amiti, M.-E.; Latkin, C. The Role of Social Networks and Geography on Risky Injection Behaviors of Young Persons Who Inject Drugs. *Drug and Alcohol Dependence* **2015**, *154*, 229–235, doi:10.1016/j.drugalcdep.2015.06.042.
8. Temple, R. Enrichment of Clinical Study Populations. *Clin Pharmacol Ther* **2010**, *88*, 774–778, doi:10.1038/clpt.2010.233.
9. Kerr, K.F.; Roth, J.; Zhu, K.; Thiessen-Philbrook, H.; Meisner, A.; Wilson, F.P.; Coca, S.; Parikh, C.R. Evaluating Biomarkers for Prognostic Enrichment of Clinical Trials. *Clin Trials* **2017**, *14*, 629–638, doi:10.1177/1740774517723588.
10. Irazabal, M.V.; Abebe, K.Z.; Bae, K.T.; Perrone, R.D.; Chapman, A.B.; Schrier, R.W.; Yu, A.S.; Braun, W.E.; Steinman, T.I.; Harris, P.C.; et al. Prognostic Enrichment Design in Clinical Trials for Autosomal Dominant Polycystic Kidney Disease: The HALT-PKD Clinical Trial. *Nephrology Dialysis Transplantation* **2017**, *32*, 1857–1865, doi:10.1093/ndt/gfw294.
11. Ferguson, T.S. Who Solved the Secretary Problem? *Statistical Science* **1989**, *4*, 282–289, doi:10.1214/ss/1177012493.
12. Camidge, D.R.; Park, H.; Smoyer, K.E.; Jacobs, I.; Lee, L.J.; Askerova, Z.; McGinnis, J.; Zakharia, Y. Race and Ethnicity Representation in Clinical Trials: Findings from a Literature Review of Phase I Oncology Trials. *Future Oncology* **2021**, *17*, 3271–3280, doi:10.2217/fon-2020-1262.
13. Ma, M.A.; Gutiérrez, D.E.; Frausto, J.M.; Al-Delaimy, W.K. Minority Representation in Clinical Trials in the United States: Trends Over the Past 25 Years. *Mayo Clinic Proceedings* **2021**, *96*, 264–266, doi:10.1016/j.mayocp.2020.10.027.
14. Kennedy-Martin, T.; Curtis, S.; Faries, D.; Robinson, S.; Johnston, J. A Literature Review on the Representativeness of Randomized Controlled Trial Samples and Implications for the External Validity of Trial Results. *Trials* **2015**, *16*, 495, doi:10.1186/s13063-015-1023-4.
15. Cohen, J. *Statistical Power Analysis for the Behavioral Sciences*; 2nd ed.; Lawrence Erlbaum Associates, 1988;
16. Mackesy-Amiti, M.E.; Gutfraind, A.; Tatara, E.R.; Collier, N.T.; Cotler, S.J.; Page, K.; Ozik, J.T.; Boodram, B.; Major, M.E.; Dahari, H. Simulations of HCV Vaccine Trials Demonstrate Effects of

Background Incidence and Unbalanced Exposure That Can Impact Vaccine Efficacy.; Hepatology, October 1 2021; Vol. 74, p. 604A.
17. Friede, T.; Pohlmann, H.; Schmidli, H. Blinded sample size reestimation in event-driven clinical trials: Methods and an application in multiple sclerosis. *Pharmaceutical Statistics* **2019**, *18*, 351–365, doi:10.1002/pst.1927.
18. Stark, M.; Hesse, M.; Brannath, W.; Zapf, A. Blinded Sample Size Re-Estimation in a Comparative Diagnostic Accuracy Study. *BMC Medical Research Methodology* **2022**, *22*, 115, doi:10.1186/s12874-022-01564-2.
19. Venn, M.L.; Knowles, C.H.; Li, E.; Glasbey, J.; Morton, D.G.; Hooper, R.; ESCP EAGLE Safe Anastomosis Collaborative Implementation of a Batched Stepped Wedge Trial Evaluating a Quality Improvement Intervention for Surgical Teams to Reduce Anastomotic Leak after Right Colectomy. *Trials* **2023**, *24*, 329, doi:10.1186/s13063-023-07318-9.
20. Yao, X.; Attia, Z.I.; Behnken, E.M.; Walvatne, K.; Giblon, R.E.; Liu, S.; Siontis, K.C.; Gersh, B.J.; Graff-Radford, J.; Rabinstein, A.A.; et al. Batch Enrollment for an Artificial Intelligence-Guided Intervention to Lower Neurologic Events in Patients with Undiagnosed Atrial Fibrillation: Rationale and Design of a Digital Clinical Trial. *American Heart Journal* **2021**, *239*, 73–79, doi:10.1016/j.ahj.2021.05.006.
21. Wermuth, P. Participant Recruitment, Screening, and Enrollment. In *Principles and Practice of Clinical Trials*; Piantadosi, S., Meinert, C.L., Eds.; Springer International Publishing: Cham, 2022; pp. 257–278 ISBN 978-3-319-52636-2.
22. Breiman, L. Random Forests. *Machine Learning* **2001**, *45*, 5–32, doi:10.1023/A:1010933404324.
